# Advances in Nanoparticles in the Prevention and Treatment of Myocardial Infarction

**DOI:** 10.3390/molecules29112415

**Published:** 2024-05-21

**Authors:** Qidong Wei, Yifei Xiao, Lixin Du, Ya Li

**Affiliations:** College of Pharmacy, Hunan University of Chinese Medicine, Changsha 410208, China; wqd20233763@stu.hnucm.edu.cn (Q.W.); 20213659@stu.hnucm.edu.cn (Y.X.); 20223723@stu.hnucm.edu.cn (L.D.)

**Keywords:** myocardial infarction, nanoparticles, prevention, treatment

## Abstract

Myocardial infarction (MI) is one of the most prevalent types of cardiovascular disease. During MI, myocardial cells become ischemic and necrotic due to inadequate blood perfusion, leading to irreversible damage to the heart. Despite the development of therapeutic strategies for the prevention and treatment of MI, their effects are still unsatisfactory. Nanoparticles represent a new strategy for the pre-treatment and treatment of MI, and novel multifunctional nanoparticles with preventive and therapeutic capabilities hold promise for the prevention and treatment of this disease. This review summarizes the common types and properties of nanoparticles, and focuses on the research progress of nanoparticles for the prevention and treatment of MI.

## 1. Introduction

According to the World Health Organization, ischemic heart disease is the leading cause of death worldwide, and MI is its most serious clinical manifestation of ischemic heart disease [1]. How to better prevent the occurrence of MI as well as timely and effective treatment are particularly important.

Thrombolytic therapy or direct percutaneous coronary intervention in time after the occurrence of MI, followed by the use of lipid-lowering drugs and anti-coagulants, are the most effective treatment interventions for patients with MI, which can significantly reduce myocardial infarction size and mortality [2,3]. Percutaneous coronary intervention can restore the blood flow of the infarcted coronary artery, but it is prone to ischemia-reperfusion (I/R) injury, including further tissue damage and myocardial cell apoptosis, which will eventually lead to the development of heart failure and other poor prognosis [4,5]. In addition, the body will undergo some pathological changes after MI, such as reactive oxygen species (ROS) production, mitochondrial dysfunction, calcium overload, and other mechanisms that induce cell death and inflammatory response [6]. Although anti-inflammatory and anti-oxidant drugs have been found, these drugs lack targeting, low bioavailability, and short half-life, which cannot achieve the desired efficacy [7]. Therefore, there is an urgent need to develop safer and more effective strategies for the prevention and treatment of MI.

**Figure 1:**
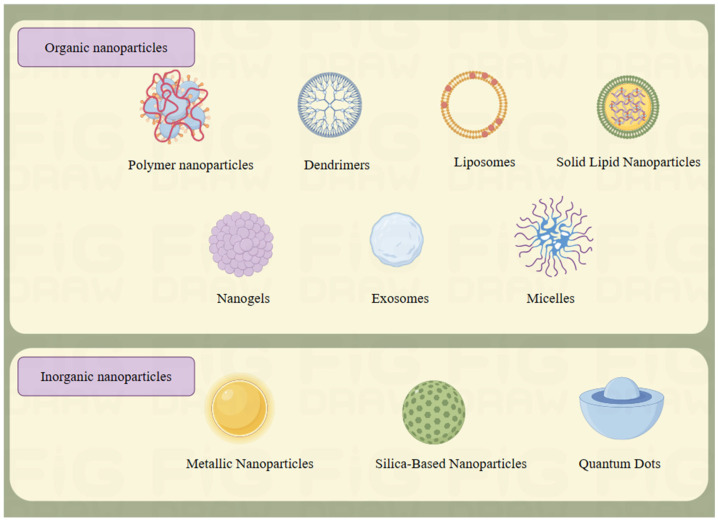
Types of nanoparticles. This figure was drawn using figdraw (https://www.figdraw.com, accessed on 29 January 2024), export ID: PARUO46dcd.

Nanoparticles are defined as particles with a size range between 1–100 nm. They are widely used in medicine due to their many different structures, sizes, shapes, surface chemistry, and other properties [8]. Nanodelivery systems have many advantages over conventional drug delivery systems, such as reduced side effects, increased bioavailability, improved drug efficacy, reduced drug dosage, longer administration time, altered routes of administration, and reduced therapeutic costs [9]. This review summarizes the physicochemical properties of common carriers, such as polymer nanoparticles, dendrimers, micelles, nanogels, liposomes, and solid lipid nanoparticles (Figure 1), and describes the development and application of some of these nanoparticles in preventing and treating MI according to its pathological mechanisms.

## 2. Pathologic Mechanisms of MI

### 2.1. Pathophysiology of MI

At present, MI is divided into five types: type 1, 2, 3, 4 and 5, among which type 1 and type 2 are the most common. Type 1 MI is mostly caused by coronary artery thrombosis caused by atherosclerosis (AS) plaque rupture (rupture/erosion), which leads to myocardial ischemia and hypoxia. The pathophysiological mechanisms that lead to ischemic myocardial injury in the presence of an imbalance between oxygen supply and demand are classified as type 2 MI [10]. Previous studies have shown that the occurrence of MI is closely related to plaque breakage in AS [11]. MI includes ST-segment Elevation Myocardial Infarction (STEMI) and Non-ST-Segment Elevation Myocardial infarction (NSTEMI) [12]. STEMI is caused by the exposure of the necrotic core after the rupture of the coronary lipid plaque, which induces the formation of an acute occlusive thrombus and ischemia of the entire myocardial layer, resulting in ST-segment elevation [13]. Compared with STEMI, NSTEMI mainly induces acute non-occlusive thrombosis on the basis of coronary plaque injury, or occlusive thrombosis with good collateral circulation, which manifests as ST-segment depression on an electrocardiogram [14]. In one study, it was found that patients with STEMI were mainly caused by plaque rupture, whereas patients with NSTEMI were mainly caused by plaque erosion [12,15]. In plaque erosion, most of them are composed of fibrous plaques with stable structure, and the degree of luminal stenosis caused by eroded plaques is lower. Therefore, for patients with NSTEMI, anti-thrombotic therapy is more preferred than direct percutaneous coronary intervention.

After the occurrence of MI, promoting the restoration of blood flow in ischemic myocardium may cause myocardial reperfusion injury, leading to myocardial cell death, including apoptosis, necrosis, and pyroptosis [16]. Apoptosis is a kind of active physiological death of cells, which can occur through intrinsic mitochondrial pathway and extrinsic death receptor pathway [17]. Cardiomyocyte apoptosis can occur through a variety of pathways. Mitochondrial dysfunction is one of the main causes of cardiomyocyte apoptosis, which can lead to the release of a large number of ROS and Ca^2+^, and can also release cytochrome C, which binds to apoptotic protease activator. In addition, it can affect the cellular physiological processes such as traditional calcium metabolism and energy metabolism, resulting in cell death [18]. After MI, the generation of ROS increases, and the activities of superoxide dismutase (SOD) and glutathione peroxidase (GSH-Px) in the body decrease, which destroys the structure of myocardial cells, damages the membrane of myocardial cells, leads to the necrosis of myocardial cells, and releases myocardial markers into the blood [19]. In the absence of cysteinase 8, receptor-interacting serine/threonine protein kinases (RIPK)-1 and -3 and mixed lineage kinase domain-like protein (MLKL) will form necrosomes, and the cell death signal will switch from apoptosis to necrosis [20]. RIPK3 phosphorylates MLKL in the necrosomes, prompting its oligomerization reaction and localization to the cell membrane, leading to burst cell membrane and inducing inflammatory response [21]. As a new pro-inflammatory programmed cell death mode, pyroptosis is characterized by caspase-1 dependence and the release of a large number of pro-inflammatory factors. It is obviously different from apoptosis in cell morphology and mechanism. When pyroptosis occurs, numerous 1–2 nm pores are formed on the cell membrane, which causes the cell membrane to lose its integrity, releases cell contents, and induces an inflammatory response [22]. In addition, reperfusion injury causes damage to the coronary microvasculature. Microembolism occurs when particulate fragments from ruptured epicardial plaques fall off into the microcirculation. In addition, even if the infarct-related coronary arteries are opened timely by percutaneous coronary intervention, the ischemic myocardium sometimes cannot receive effective blood perfusion, resulting in endothelial cell damage and no-reflow phenomenon [23]. In addition, some studies have found that early reperfusion will lead to aggravation of coronary endothelial cell damage, expansion of infarct size and deterioration of cardiac function [24].

### 2.2. Heart Repair in MI

**Figure 2:**
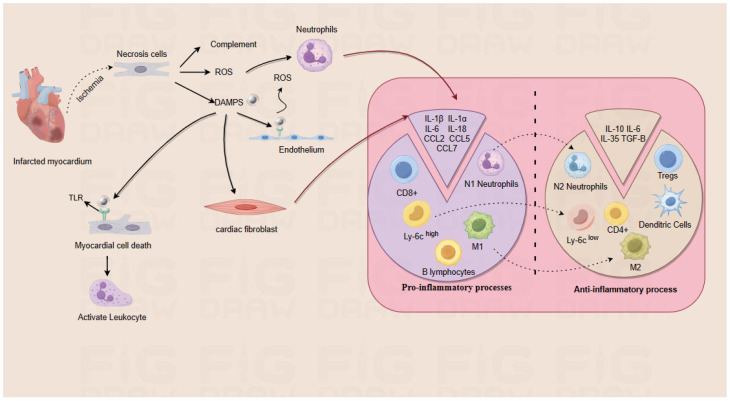
Overview of the inflammatory response to acute myocardial infarction. The figure outlines the pro-inflammatory and anti-inflammatory phases after MI. Myocardial ischemia leads to cell necrosis after MI. Necrotic cardiomyocytes induce inflammation by producing complement, ROS, and DAMPS. Complement induces the aggregation of cells, such as neutrophils, B lymphocytes, and macrophages, to the infarcted myocardium by releasing related cytokines. A variety of DAMPS can be produced by cardiac resident necrotic cells after myocardial infarction. DAMPS can bind to Toll-like receptors on endothelial cells and cardiomyocytes, promote ROS production by endothelial cells, and also cause cardiomyocyte necrosis and activate leukocytes. In addition, DAMPS are able to stimulate the release of inflammatory cytokines and chemokines from fibroblasts. In the anti-inflammatory stage, monocytes, neutrophils, and macrophages will undergo changes and work with Tregs, MDSC, and dendritic cells to secrete anti-inflammatory cytokines to resolve the inflammatory response, so that the damaged heart will enter the next repair stage. This figure was drawn using figdraw (https://www.figdraw.com, accessed on 20 March 2024), export ID: RUAOP72570.

The MI heart undergoes pro-inflammatory and anti-inflammatory processes (Figure 2) to promote wound healing and scar formation, thereby preventing cardiac rupture, which can be subdivided into three phases: inflammatory phase, repair proliferative phase, and maturation phase. The first phase is the inflammatory phase, during which dead cells and extracellular matrix debris in the infarcted area are cleared through an inflammatory response [25]. During this phase, damaged or necrotic cells and the damaged extracellular matrix release danger signals called damage-associated molecular pattern (DAMP), which bind to pattern recognition receptors expressed by surviving parenchymal cells and other cells of the innate immune system, activating a series of inflammatory mediators that stimulate a systemic inflammatory response, including inflammatory factors, chemokines, elevated leukocytes, and cell adhesion molecules [26]. The second phase is the repair proliferation phase, which focuses on inhibiting the inflammatory response and synthesizing myofibroblasts to achieve repair and maintain the structural integrity of the infarcted heart [26]. The inhibition of the inflammatory response, at the beginning, is mainly through the processes of pro-inflammatory M1-type macrophages switching to anti-inflammatory M2-type macrophages. In this phase, phagocytes exert a cytosolic role, and secretion occurs of anti-inflammatory cytokines by subpopulations of T-lymphocytes [27]. Meanwhile, the adult mammalian heart contains a large number of mesenchymal and perivascular fibroblasts, which transdifferentiate into myofibroblasts and secrete matrix proteins, and myofibroblasts express contractile proteins (e.g., α-smooth muscle actin), which repair the infarcted myocardium and promote the formation of scar tissue [28]. In addition, when matrix fragments in the infarcted area are phagocytosed, the organism forms a temporary matrix composed mainly of fibronectin and fibronectin, and this highly plastic matrix network serves as a scaffold for cell migration and proliferation to promote wound healing [26]. The last stage is the maturation phase, which concludes the healing process of the infarct by altering the cellular composition of the scar tissue and the composition of the extracellular matrix. For example, the number of activated myofibroblasts in the infarcted area decreases. Post-infarct myofibroblasts are in a quiescent state and may undergo apoptosis or show diminished expression of contractile proteins [29].

## 3. Types and Properties of Nanoparticles

### 3.1. Organic Nanoparticles

#### 3.1.1. Polymer Nanoparticles

Polymer nanoparticles are derived from a range of natural or synthetic polymeric materials, and their particle size and surface charge vary depending on the specific type of polymer [30]. Polymers can be classified into non-biodegradable and biodegradable types. Current research focuses on biodegradable polymers due to concerns that non-biodegradable polymers may lead to inflammatory reactions and chronic toxicity [31]. Biodegradable polymers can be either synthetic or natural. Natural biodegradable polymers are widely utilized in nanodrug delivery systems because of their biodegradability, biocompatibility, low toxicity, good stability, natural renewability, and low cost [32,33]. The polymers can be derived from animals, plants, fungi, and bacteria and are mainly polysaccharide and protein-based polymers [34]. Their portfolio of polysaccharide-based polymers includes chitosan, hyaluronic acid, alginate, and dextran, while their protein-based polymers include zein, gelatin, and albumin [35]. Synthetic biodegradable polymers can be designed according to different needs such as different drugs, disease targets, degradation rates, and delivery routes. The common synthetic biodegradable polymers are poly(lactic-co-glycolic acid) (PLGA), poly(epsilon caprolactone) (PCL), and poly(lactic acid) (PLA) [32]. Ikeda et al. [36] combined PLGA-mediated cyclosporine A (CsA) and pitavastatin in a mouse model of ischemia-reperfusion (I/R) injury. The combination of the two nanoparticles was found to inhibit mitochondrial permeability transition pore opening, reduce monocyte-mediated inflammation, and protect the damaged heart.

Although both biodegradable polymers have their own advantages, they also have their own drawbacks. For example, natural biodegradable polymers are capable of causing disadvantages such as immune rejection in vivo and high variability between batches; synthetic biodegradable polymers may cause cytotoxicity and immunogenicity during degradation in vivo [32].

#### 3.1.2. Dendrimers

Dendrimers are synthetic polymers, also known as starburst polymers, that were developed in the 1980s [37]. They consist of three parts: a central core, branched chains, and terminal functional groups, and have abundant intramolecular voids with surface modifiability [38]. Drug delivery is carried out through two binding modes: non-covalent interactions (the dendritic polymer wraps the drug molecule inside the dendritic polymer) or covalent interactions (the drug is covalently linked to the dendritic polymer) to enhance drug solubility, absorption, stability, bioavailability, and targeting distribution [39,40]. However, due to the molecular cytotoxicity of some cationic terminal functional groups in higher generations of dendrimers, in order to reduce their toxicity, researchers have generally reduced the surface cationic terminal functional groups or introduced different chemical modifications at the periphery [37,40]. Polyamide-amine dendrimers are the most thoroughly studied dendrimers and were the first nanoscale dendrimers to be chemically synthesized, characterized, and commercialized [41]. Polyamide amine dendrimers have been shown to improve solubility, stability, and oral bioavailability of various drugs, as well as increase transdermal penetration and specific drug targeting, and are often used alone as standalone drug or gene delivery systems [39]. In gene delivery, Xue et al. [42] loaded microRNA-1 inhibitors onto dendritic polymers to inhibit apoptosis of cardiomyocytes in MI hearts. The nanodelivery group showed a significant reduction in the number of cardiomyocyte apoptosis and a reduction in the area of myocardial infarction compared to the myocardial infarction control group.

#### 3.1.3. Micelles

Polymeric micelles are colloidal dispersions of amphiphilic compounds, generally used for the delivery of hydrophobic drugs, and are between 10–100 nm in size. According to the different polymers and solutions, they can be divided into double-block, triblock, multi-block copolymers, graft polymers, stimulus-sensitive polymers, etc. The amphiphilic block copolymer, for example, is usually self-assembled in aqueous solution and is a copolymer with a hydrophobic core and a hydrophilic shell [43]. Among them, the hydrophobic core plays a key role in the dissolution of hydrophobic compounds, and the compatibility between the hydrophobic core and drug molecules also determines the drug loading ability of the core. However, the presence of a hydrophilic shell can avoid clearance by the reticuloendothelial system [44,45]. Therefore, good core-shell structure, kinetic stability and solubility of hydrophobic drugs can be considered advantages of micelles [46]. Tesoro et al. [47] used micelles to deliver anti-inflammatory interleukin-10 (IL-10) to mouse and porcine models of I/R injury and found that the nanoparticles reduced interstitial fibrosis. It also improved the left ventricular ejection fraction (LVEF) and promoted the expression of anti-inflammatory cytokines.

#### 3.1.4. Nanogels

Nanogels are colloidal systems classified as containing small nanoparticles (about 20–200 nm) and consist of cross-linked, water-swellable 3D polymer networks capable of absorbing large amounts of water or physiological fluids without dissolving [48,49]. Depending on the active substance and the type of nanogel, different modalities have been developed to facilitate drug loading into nanogels. The first is drug permeation into the nanogel according to osmotic pressure and concentration gradients and through drug–polymer interactions. The second method is to encapsulate the drug in the gel by trapping. The third is that the drug is connected to the nanogel by chemical bonds [50]. Compared with other drug delivery systems, nanogels are able to reduce side effects, reduce adverse reactions, prolong serum half-life, and reduce the risk of immune reactions [49]. Zhou et al. [51] injected the hydrogel prepared by melanin nanoparticles and alginate into the myocardium of rats with MI to study cardiac repair, and the results showed that the hydrogel could reduce the oxidative stress damage of cardiomyocytes caused by ROS. In addition, it also regulates the expression of phosphoinositide 3-kinase (PI3K)/protein kinase B (Akt)/mammalian target of rapamycin (mTOR) signaling pathway, which induces the polarization of M2 macrophages and promotes the repair of injured myocardium. At present, nanogels have been applied in various aspects, such as drug delivery, diagnosis, biosensing, and biological substance separation [52].

#### 3.1.5. Liposomes

Liposomes are self-assembled drug vesicles in an aqueous solution. As a drug delivery system, it has excellent biocompatibility and physical and chemical properties, which can effectively prevent the decomposition of the encapsulated drug in vivo, prolong the half-life of the drug, control the release of the drug, and improve the safety of the drug. At the same time, this system can also achieve specific targeting of lesions, reduce toxic and side effects, increase the drug tolerance dose, and improve efficacy [53]. Liposomes can be classified as either single-layered vesicles or multi-layered vesicles. Unilamellar vesicles are characterized by a monolipid bilayer with a diameter of 20 to 250 nm and a large aqueous core at the center suitable for encapsulation of hydrophilic drugs or antigens. Multilayer vesicles are characterized by the presence of two or more concentric lipid bilayers with a diameter of 1–5 μm, which preferentially trap lipid-soluble molecules [54]. In addition, multiple drugs can be loaded in two or more multilayer liposomes of a single liposome, so that different drug molecules can be dissociated from the shell layer to the inner core layer in a certain order [55]. In order to avoid uptake by phagocytes and reduce the half-life of blood circulation, inert polymer molecules such as polyethylene glycol (PEG) can be coated on the surface of liposomes to overcome this limitation [56]. Scalzo et al. [57] developed the delivery of plasmid DNA (pDNA) by liposomes. The results of in vitro and in vivo studies showed that liposomes could deliver pDNA to cardiomyocytes and improve the transfection efficiency of cardiomyocytes. In addition, as the only nanodrug delivery systems approved by the US Food and Drug Administration (FDA) for clinical use, it has been applied in the fields of cancer treatment, vaccines, medical diagnosis, and drug delivery [56,58,59].

#### 3.1.6. Solid Lipid Nanoparticles

Solid lipid nanoparticles are a colloidal carrier system composed of physiological lipids, surfactant, and water that are solid at both room temperature and body temperature. The stability can be improved by adjusting the concentration of surfactant in the range of 0.5–5% [60,61]. As an alternative system to carriers such as liposomes and polymer nanoparticles, solid lipid nanoparticles combine the advantages of polymer nanoparticles and liposomes, and are an innovative drug delivery carrier [62]. Compared to other encapsulation systems, solid lipid nanoparticle delivery systems have the advantages of controlled drug release, improved stability of active substances, and encapsulation of lipophilic or hydrophilic drugs, which can combine lipophilic/hydrophilic components together and avoid the use of organic solvents [60,63]. Guo et al. [64] co-loaded puerarin and tanshinone in solid lipid nanoparticles to study the therapeutic effect on rats with MI. The solid lipid nanoparticle group loaded with both drugs had the smallest myocardial infarct size and high healing compared to the other control groups. In addition to conventional oral delivery, solid lipid nanoparticles can be used as carriers for intranasal, dermal, ocular, and pulmonary delivery [65].

#### 3.1.7. Exosome

Exosomes are a class of extracellular vesicles with diameters ranging from 40–160 nm, and they are produced in multivesicular bodies. It contains a variety of substances, such as proteins, lipids, metabolites, DNA, non-coding RNA, etc., and also participates in the regulation of various physiological and pathological processes of host cells [66]. It is known that exosomes can be secreted by most cells, including immune cells, cancer cells, endothelial cells, and mesenchymal stem cells [67,68,69,70]. In addition, exosomes are widely present in various body fluids, such as blood, amniotic fluid, urine, cerebrospinal fluid, saliva, etc. [71]. As a new type of carrier, exosomes have the advantages of less toxicity, being able to cross biological barriers and avoid phagocytosis by mononuclear phagocytic system compared with synthetic nanoparticles [72]. Zhu et al. [73] used exosomes derived from adipose-derived stem cells to deliver microRNA-31 to promote cardiac angiogenesis in MI mice. Nowadays, exosomes have been applied in many fields, such as cancer monitoring after radiotherapy and the treatment of ventricular remodeling after MI, and exosomes for the treatment of cancer have also been approved for a number of clinical trials [74,75,76].

### 3.2. Inorganic Nanoparticles

#### 3.2.1. Metal Nanoparticles

Metal nanoparticles mainly refer to nanoparticles with a diameter of 1–100 nm prepared from metal substances, such as gold, silver, iron, and nickel, or their oxides [77]. The surface of metal nanoparticles can be functionalized and conjugated with anti-bodies, ligands, drugs, etc., to realize their applications in biotechnology, drug and gene delivery, imaging, and other fields [78]. The surface of metal nanoparticles is easy to interact with targeting agents and other molecules through hydrogen bonding, covalent bonding, and electrostatic interactions, and shows higher stability and longer half-life in terms of circulation, biodistribution, and targeting target sites [79]. Arozal et al. [80] used a rat model of MI induced by isoproterenol (Iso) to evaluate the protective effect of silver nanoparticles on myocardial infarction. The results showed that silver nanoparticles could reduce the level of malondialdehyde (MDA) and the expression of nuclear factor kappa-B (NF-κB) protein in the heart tissue, thereby protecting the myocardium from MI. In addition, metal nanoparticles have unique physical properties, such as magnetic properties, that can be used for clinical diagnosis. At present, superparamagnetic iron oxide nanoparticles (SPION) are often used as contrast agents for magnetic resonance imaging in clinical practice [81].

#### 3.2.2. Silica Nanoparticle

Silica nanoparticle is a kind of crystalline particles composed of silica with a regular structure. The siloxane structure and silanol group of these nanoparticles make it easy to bond with a variety of biomolecules to achieve the desired purpose [82]. There are two main types of silica: solid silica nanoparticles (SSN) and mesoporous silica nanoparticles (MSN). MSN has pores in the range of 2–50 nm, which can be modified to have a high specific surface area [83]. In recent years, silica nanoparticles have been widely concerned because of their many advantages. This nanoparticle has shown great application potential in many fields due to its unique morphology, pore structure, porosity, good chemical stability, and good physical and chemical properties [83]. Yu et al. [84] combined microRNA-24, an inhibitor of cardiomyocyte apoptosis, with silica nanoparticles and injected it into the edge of the infarct area in a rat model of MI. The in vivo study showed that the nanoparticles could effectively inhibited cardiomyocyte apoptosis, thereby improving ventricular remodeling and cardiac function. It should also be noted that the size of silica has a great relationship with the indication. It is suitable for imaging and other applications for silica nanoparticles with a diameter of less than 10 nm. Those in the 50–300 nm diameter range can be used for drug delivery applications; those larger than 300 nm in diameter can be used for experimental oral drug delivery [85].

#### 3.2.3. Quantum Dots

Quantum dots are semiconductor nanocrystals with a spherical core-shell structure. When the semiconductor nanocrystalline size is in the characteristic length range of 2–10 nm, the particle acquires unique electrical and optical properties [86]. Compared with the common traditional organic fluorophores, quantum dots have the characteristics of broad excitation spectrum, narrow fluorescence emission spectrum, detection sensitivity, and easy biological coupling [87]. Based on these characteristics, quantum dots have been applied in different fields, such as optoelectronic devices, biological imaging, and solar energy devices [88]. In the medical field, quantum dots can be used both as nanoprobes for imaging and for therapy, such as photosensitizers for photodynamic therapy or photothermal agents for photothermal therapy [89]. In the diagnosis of MI, Tabish et al. [90] used a graphene quantum dot electrochemical biosensor for the early diagnosis of MI, which was experimentally proved to have high sensitivity, specificity, and selectivity for detecting cardiac troponin and myoglobin.

## 4. Nanoparticles for MI Prevention

AS is a chronic inflammatory disease characterized by the accumulation and deposition of cholesterol in arteries and remodeling of the extracellular matrix in the intima and inner media. The inflammation of endothelial cells, proliferation of vascular smooth muscle cells (SMC), and recruitment of monocytes and macrophages play a key role in the development of AS. AS is the main cause of MI, and about 70% of MI is caused by AS plaque rupture [91]. Studies have shown that inflammation, hyperlipidemia, hyperglycemia, hypertension, and clonal hematopoiesis are the main causes of AS plaque [92]. Therefore, the development of corresponding nanoparticles to reduce the risk of plaque formation or rupture according to the reasons mentioned above is of great significance for the prevention of MI; see Table 1.

Wei et al. [93] proposed a nanostructure based on non-cationic nucleic acids, with a core of PEG-coated SPION and a shell containing a layer of phosphorothioate (PS)-modified microRNA-146a (miR-146a) oligonucleotide. These oligonucleotides were linked to PEG-coated SPION. An miR-146a functionalized SPION (Mir-146a-SPION) spherical nucleic acid (SNA) nanostructure was obtained. By intravenous injection into apolipoprotein E knockout (ApoE−/−) mice fed a high-cholesterol diet, it can be abundantly injected into macrophages and endothelial cells without the help of cationic or lipophilic transfectants to inhibit NF-κB signaling. The results showed that the nanoparticles were able to reduce inflammation and reduce and stabilize plaques without causing severe toxicity. Because most MI is caused by AS plaque rupture, He et al. [94] used simvastatin as a model statin to inhibit plaque inflammation and promote cholesterol efflux, and designed nanoparticles consisting of β-cyclodextrin-locked discoidal recombinant high-density lipoprotein and a hyaluronic acid-ferrocene conjugate. The mice were injected into the AS model by tail vein injection. Compared with the saline group, the nanoparticle group showed a 53% reduction in plaque size, 63% reduction in plaque lipid deposition, 62% reduction in plaque macrophage content, and 64% reduction in local inflammatory factor levels. At the same time, the nanoparticles also alleviated systemic inflammation characterized by reduced levels of serum inflammatory factors.

**Table 1:** Application of nanoparticles in the prevention of MI.

Types of Nanoparticles	Payload	Target	Animal Model	Outcomes	Ref.
PLGA nanoparticles	siCamk2g	Macrophages	LDLR−/−mice	Necrotic core area decreased, fibrous cap thickness increased, and plaque stability increased	[95]
Macrophage membrane-coated PLGA nanoparticles	Rapamycin	AS plaque	ApoE−/−mice	Plaque lipids were reduced and necrotic core area was reduced	[96]
Macrophage membrane-coated nanoparticles	Atorvastatin	ROS	ApoE−/−mice	Plaque area and necrotic core area were significantly reduced	[97]
PLGA nanoparticles	Colchicine	AS plaque	ApoE−/−mice	Plaque lipid deposition was reduced and plaque area was reduced	[98]
Lipid nanoparticles	Apolipoprotein A-I	AS plaque	LDLR−/−mice	Inhibits the inflammatory response of macrophages, inhibits AS, and stabilizes AS plaques	[99]
Erythrocyte membrane-coated stellate polymers	Probucol	ROS	ApoE−/−mice	The formation of foam cells was inhibited and the plaque area was reduced	[100]
Mannose-modified dendritic nanoparticles	SR-A siRNA and LXR ligands	Macrophages	LDLR−/−mice	Plaque area was reduced and aortic cholesterol content was reduced	[101]
Hyaluronic acid-modified liposomes	Rosuvastatin	AS plaque	ApoE−/−mice	The levels of proinflammatory factors and foam cells were reduced, and plaque area was reduced	[102]
Micellar nanoparticles	microRNA-145	Vascular smooth muscle cells	ApoE−/−mice	Plaque size and overall lesion area were reduced	[103]
Macrophage membrane-coated nanoparticles	Methotrexate	AS plaque	ApoE−/−mice	Plaque area and plaque lipid deposition were reduced	[104]

## 5. Treatment of MI Nanoparticles

Nanocarriers can reach specific tissues or cells through enhanced permeability and retention effect (EPR) or binding to anti-bodies, proteins, and nucleic acid aptamers, so as to improve the bioavailability of encapsulated drugs and reduce their toxic side effects. Leukocytes such as mononuclear phagocytes, neutrophil packages, and T cells are present in the healthy heart. It also contains immune cells such as macrophages, dendritic cells, and lymphocytes [105]. After infarction, these cells gather to the heart from all parts of the body and produce related factors (such as ROS production by neutrophils after infarction) that disrupt the homeostasis of the internal environment in the heart [106]. This review will be described in terms of regulating cardiac homeostasis and promoting the recovery of damaged cardiomyocytes. Nanoparticles for the treatment of MI by active targeting are shown in Table 2 and Table 3.

### 5.1. Nanoparticles for Regulating Cardiac Homeostasis

PLGA has been approved by the FDA as a material for drug engineering and is highly biodegradable and biocompatible. It is the most commonly used biodegradable polymer for the preparation of nanoparticles [125]. The mitochondrial membrane potential is damaged during myocardial I/R, and Szeto-Schiller 31 (SS31) is a synthetic peptide that specifically binds to mitochondrial inner phospholipids, so this property can be used for targeted drug delivery [126]. Zhang et al. [126] combined the mitochondrial targeting peptide SS31 with PLGA and PEG to prepare mitochondrial targeting nanoparticles (CsA@PLGA-PEG-SS31) by precipitation. In the rat I/R model, intravenous injection of CsA@PLGA-PEG-SS31 was shown to increase CsA delivery to cardiomyocytes. TTC/Evans blue staining of heart tissue showed that the percentage of infarct size was 46% in the model group and 19% in the CsA@PLGA-PEG-SS31 treatment group, indicating that CsA@PLGA-PEG-SS31 exhibited cardioprotective effects, reduced apoptosis and reduced cardiac remodeling in rats with myocardial reperfusion.

After the occurrence of MI, neutrophils first reach the infarct area and produce ROS to induce inflammation [106]. Studies have found that natural products show certain anti-inflammatory effects both in vivo and in vitro. For example, ginsenoside Rg3 (Rg3) is the main active ingredient of ginseng, which has a series of pharmacological effects including anti-oxidation, anti-inflammation, and anti-aging [127]. Li et al. [127] used a diblock copolymer of PEG and polypropylene thioethers (PPS) to encapsulate Rg3 to form ROS-responsive polymer nanoparticles (PEG-B-PPS-Rg3). By intra-myocardial injection of PEG-b-PPS-Rg3 into the rat I/R model, it was found that Rg3-loaded PEG-b-PPS nanoparticles responded to ROS. The released Rg3 interacted with the target protein FoxO3a to alleviate myocardial I/R injury and activate its downstream signaling pathways. Oxidative stress, inflammation, apoptosis and fibrosis are inhibited to achieve effective treatment of myocardial I/R.

In addition to conventional therapies, RNA interference (RNAi) is a promising therapeutic method that can achieve the precise release of RNAi delivery in vivo. Because microRNA (miRNA) can stimulate cardiac function, Li et al. [128] used mesoporous silica nanoparticles as miRNA delivery vectors. They combined the anti-inflammatory material MSN with pro-angiogenic miR-21-5p to form the MSN/miR-21-5p complex, which was embedded in an injectable hydrogel matrix to form Gel@MSN/miR-21-5p complex. After the porcine MI model was completed, the Gel@MSN/miR-21-5p complex was injected into the middle myocardial layer of 2 × 2 cm by injection. It was found that the complex could inhibit the transcription factor NF-κB and Toll-like receptor signaling pathways, activate the vascular endothelial growth factor and extracellular signal-regulated kinase/mitogen-activated protein kinase pathways, thereby improving myocardial remodeling, reducing myocardial fibrosis, reducing myocardial infarction size, and promoting angiogenesis.

In recent years, the emergence of biomimetic nanotechnology has provided a new strategy for the treatment of MI. Biomimetic nanotechnology combines cell membranes or extracellular vesicles with nanoparticles [129]. This not only preserves the physicochemical properties of nanoparticles, but also takes advantage of the complex cellular functions [130]. Such nanoparticles combined with biomaterials can achieve immune escape, prolong systemic circulation time, and precisely target disease sites [131]. Previous studies have shown that extracellular vesicles can not only serve as transport vehicles, but also serve as cell-free therapy for MI due to their cardioprotective effects such as increasing the survival rate of cardiomyocytes, inhibiting inflammatory response and oxidative stress [132]. Curcumin has been shown to be beneficial in myocardial infarction [133]. Ji-Young et al. [134] curcumin was loaded with miR-144-3p into extracellular vesicles modified with a heart-targeting peptide and then injected intravenously into mice with MI. It was found that the extracellular vesicles could improve the accumulation and bioavailability of curcumin in the heart, and reduce the infarct size and cardiomyocyte death after MI. Among the cell membrane-coated nanoparticles, the cell membranes of different cells have different characteristics. For example, the erythrocyte membrane has the role of immune evasion due to the CD47 membrane protein on its surface, the neutrophil membrane has the role of selective targeting of inflammatory tissues, and the platelet membrane can selectively target the compliance of inflammatory neutrophils in damaged tissues [135]. Fangyuan et al. [136] encapsulated CsA in poly (5, 5-dimethyl-4, 6-dithiopropanediolate), encapsulated in platelet membrane to form a regulatory T cell biomimetic nanoparticle, and demonstrated that this biomimetic nanoparticle can reduce the infarct size and fibrosis area, and can reduce the apoptosis of cardiomyocytes, and enhance the function of the heart in MI mice.

### 5.2. Nanoparticles to Promote the Regeneration of Damaged Hearts

After MI, myocardial cells will die irreversibly, and the ability of adult myocardial cells to proliferate is weak. The infarct area is easily replaced by scar tissue with low elasticity, resulting in heart failure. It is now possible to restore cardiac function by delivering stem cells to infarcted areas or by delivering relevant stimuli to stimulate endogenous production of cardiomyocytes, thereby ameliorating phenomena such as heart failure and ventricular remodeling.

Stem cells are widely used in the medical field due to their ability of self-renewal and differentiation into various tissues [137]. Stem cells can be divided into bone marrow stem cells, mesenchymal stem cells, and cardiac stem cells according to their sources. Among them, mesenchymal stem cells are considered as a promising therapy because of their ability to differentiate into endothelial cells and cardiomyocyte-like cells and improve cardiac function after myocardial infarction [138]. Zhu et al. [139] encapsulated mesenchymal stem cells and ^68^Ga^3+^ cations into synthetic silicate nanoplatelets, which are composed of gold nanorods (GNRs) and synthetic silicate nanoplatelets (SNs) and poly (lactide-glycolic acid copolymer)-b-poly (ethylene glycol)-b-poly (lactide-glycolic acid copolymer) (PLGA-PEG-PLGA) in the conductive nanocomposite hydrogel (MSC/GNR@SN/Gel). After injection of MSC/GNR@SN/Gel into the infarcted myocardium of mice, it was found that MSC/GNR@SN/gel could enhance the mechanical strength of myocardium. Compared with other MI groups, MSCs/GNR@SN/Gel group had a smaller increase in left ventricular end-systolic diameter and left ventricular end-diastolic diameter, the highest left ventricular ejection fraction and left ventricular fractional shortening, a significant reduction in collagen volume fraction, and a significant increase in left ventricular wall, which could protect myocardial viability and improve ventricular remodeling.

However, the transplantation of mesenchymal stem cells (MSCs) to treat MI also has some disadvantages, such as low survival rate in vivo, potential immune rejection and the risk of tumor formation [140]. The delivery of relevant genes and stimulating factors to promote cardiomyocyte proliferation or inhibit apoptosis becomes another approach. Li et al. [141] found that polymer nanoparticles carrying miRNA can achieve local targeted delivery through shear-thinning injectable hydrogels. Their team used miR-199a-3p as a model miRNA to conduct in vitro and in vivo experiments. The results showed that the hydrogel complexes could promote the proliferation of human embryonic-stem-cell-derived cardiomyocytes and endothelial cells in vitro. Moreover, it has low cytotoxicity. In vivo studies have shown that this complex can improve the ejection fraction, reduce scar formation, and increase capillary density in the marginal region, thereby improving cardiac function and repairing the damaged heart in a mouse model of myocardial ischemia-reperfusion. In addition, reprogramming non-myocardial cells to induce cardiomyocyte-like cells for therapeutic purpose has also shown great potential in the field of myocardial regeneration. Yang et al. [142] used branched polyethylenimine coated nitrogen-rich carbon spots to load miRNA to form nanocomposites. Four genes, i.e., mirNA-1, mirNA-133, mirNA-208, and mirNA-499, were used as miRNA complexes. It was found that the nanocomposites could promote direct reprogramming of fibroblasts into cardiomyocyte-like cells and restore cardiac function in the heart.

## 6. Conclusions and Prospects

MI is usually treated with anti-thrombotic drugs, vasodilators, percutaneous coronary intervention, or coronary artery bypass grafting. However, none of these approaches could achieve the desired effect, because myocardial I/R injury will lead to the death of myocardial cells and myocardial inflammation and destroy the homeostasis of the heart. The ability of adult mammals to regenerate myocardium is insufficient to replenish the number of cardiomyocytes that die due to I/R injury, and eventually, necrotic myocardium is replaced by scar tissue, leading to cardiac insufficiency and triggering heart failure. Traditional systemic administration has the disadvantages of low bioavailability, more toxic side effects, and short systemic circulation time. Nanodrug delivery systems have great potential as carriers to protect encapsulated drugs from biodegradation and reach specific tissues or cells.

With the development of nanomaterials, the types of nanoparticles are becoming more and more abundant. When using nanoparticles to deliver relevant drugs, we should not only consider the size, shape, and uptake of nanoparticles, but also consider whether the physicochemical properties of the carrier can play a beneficial role in the disease. For example, some dendritic polymer carriers themselves have anti-fungal and anti-bacterial effects, and nanogel injection into the myocardium can play a supporting role in the damaged heart [40]. At present, nanoparticles have been used in the clinical diagnosis of MI, but few nanoparticles have been used in the clinical treatment of MI, probably because of the toxicity of the carrier itself. According to reports, about 20% of nanoparticles that enter clinical trials are rejected due to safety concerns [143]. The toxicity of nanoparticles depends on their biophysical properties, including properties such as size, surface area, and surface charge, which can affect their distribution and deposition in different organ systems and alter their molecular interactions with various proteins and other macromolecules [144]. The size of nanoparticles plays an important role in their entry route, cellular uptake, and overall toxicity. Li et al. studied the effect of the size of gold nanoparticles on biotoxicity and biodistribution, and concluded that higher concentrations and smaller gold nanoparticles could cause more cytotoxicity [145]. Nanoscale materials have a variety of shapes and structures, and different shapes may have implications for deposition kinetics and cellular uptake mechanisms. Bhattacharya et al. investigated the cytotoxicity and genotoxicity of spherical and rod-shaped Zno nanoparticles on human peripheral blood mononuclear cells and found that rod-shaped nanoparticles were more cytotoxic than spherical Zno nanoparticles [146]. Studies have shown that charged nanoparticles are better able to aggregate at the target site [147]. Nel et al. found in vitro that cationic nanoparticles functionalized on the surface were more cytotoxic than anionic or neutral nanoparticles [148]. Therefore, according to the properties of different nanomaterials, we can design less toxic nanoparticles and accelerate their clinical application.

In addition, we found that most of the animal models used in MI are rodents, and there are few studies using large animals, which is also a limitation of the study of this disease. The selection of animal models that are similar to humans in anatomy and physiology and exclude the differences between species as much as possible can provide an important theoretical basis for the prevention and treatment of MI. In terms of animal model selection, the developed MI porcine model can be selected as an MI model to provide relevant experimental data for clinical practice and verify the safety and efficacy of nanoparticles. Due to the great differences in gene expression regulation and physiological metabolism mechanism between rodents and humans, such as blood, antigen, and anti-body type, mice as animal models are less applicable to human diseases. Primates and dogs are limited by resources, ethics, and economics, and cannot be widely used. With the advantages of a small body size, high standardization, and clear biological background, pigs have gradually become an important model animal in life science research. The cardiovascular system of pigs is very similar to that of humans, especially in the aspects of electrolyte structure, diet structure, lipoprotein metabolism mechanism, system function characteristics, and drug responsiveness [149]. Therefore, pigs can be used to prepare cardiovascular disease animal models and study drug effects in the future. We also found that the most commonly used model for animal models of MI is surgical ligation of the left anterior descending artery (LAD), which can be achieved by either permanent occlusion of the LAD or IR with sutures. There are two main approaches: permanent ligation (PL), in which the LAD is permanently blocked with a suture, or IR, in which the LAD is temporarily blocked to restore blood flow and tissue reperfusion before the suture is removed. Compared with IR, PL caused a larger infarct size and was able to cause more severe damage to the myocardium. We can choose different models according to different clinical needs, and PL is directly applicable to the case of acute myocardial infarction without reperfusion therapy and the case of reperfusion therapy but progression to heart failure. Comparing the two methods, IR is more representative of human myocardial infarction than PL. For patients with myocardial infarction, the first-line treatment option is timely reperfusion therapy, which provides the opportunity to study reperfusion injury and is applicable in the case of clinical intervention in acute myocardial infarction [150].

In summary, the development of nanoparticles provides a new treatment option for MI. In the future, in order to promote the clinical application of nanoparticles in the treatment of MI and achieve the best therapeutic effect, not only more strategies to reduce the toxicity of nanocarriers, but also the development of nanodrug delivery systems with the goal of reducing myocardial cell loss, regulating myocardial remodeling or promoting myocardial regeneration, should be developed. In addition, biomimetic nanotechnology has great potential for the development of MI. The cell membrane coating of nanoparticles retains the original biological characteristics of the source cell membrane, easily crosses the biological barrier, and prolongs the circulation time in the body [151]. In the future, the cell membrane of MI-related cells can be coated with nanoparticles or scaffolds to achieve the desired prevention and treatment effect. Currently, gene therapy is a promising approach to promote the endogenous proliferation of cardiomyocytes after MI. Although viral vectors have made more progress in the treatment of MI, their further development is limited because of the immune response and toxicity of viral proteins [152]. Non-viral nanocarriers, such as liposome nanoparticles, polymer nanoparticles, and solid lipid nanoparticles, have higher safety and modifiability, which provide a better treatment for MI. Future research should be focused on these aspects, and to promote the conversion of nanoparticles into practical and effective clinical applications.

## Figures and Tables

**Table 2:** Application of nanoparticles in regulating cardiac homeostasis.

Types of Nanoparticles	Payload	Target	Animal Model	Outcomes	Ref.
Polyethylene glycol nanoparticles	Bilirubin	Infarcted myocardium	Myocardial infarction (ischemia-reperfusion, mice)	Inhibition of cell apoptosis and reduction of myocardial infarction size	[107]
Liposome nanoparticles	Methotrexate	Infarcted myocardium	Myocardial infarction (permanent occlusion, mice)	Reduced infarct size and promoted a significant improvement in left ventricular systolic function	[108]
Micellar nanoparticles	2,2,6,6-tetramethylpiperidine-1-oxyl	Infarcted myocardium	Myocardial infarction (ischemia-reperfusion, dog)	Reduced myocardial infarct size and myocardial apoptosis	[109]
PEG modification of solid lipid nanoparticles	Schisandrin B	SOD/GSH-Px	Myocardial infarction (permanent occlusion, rat)	Reduction in infarct size	[110]
PEG-graphene quantum dot nanoparticles	Curcumin	Infarcted myocardium	Myocardial infarction (permanent occlusion, rat)	Reduction of myocardial infarct size and fibrosis	[111]
Liposome nanoparticles	MI antigen and rapamycin	T cell	Myocardial infarction (permanent occlusion, mice)	Infarct size and fibrosis area were reduced	[112]
MSN conjugated to CD11b anti-body	Panax notoginseng saponins R1	Monocytes and neutrophils	Myocardial infarction (permanent occlusion, mice)	Improves local inflammation of injured myocardium and reduces myocardial infarction area	[113]
Methacrylic acid, N-isopropyl acrylamide hydrogel	Visnagin	Infarcted myocardium	Myocardial infarction (ischemia-reperfusion, rat)	Reduce myocardial infarction area and improve cardiac dysfunction	[114]

**Table 3:** Application of nanoparticles in promoting regeneration of damaged hearts.

Types of Nanoparticles	Payload	Animal Model	Outcomes	Ref.
Hyaluronic acid hydrogel	Mesenchymal stem cells	Myocardial infarction (permanent occlusion, rat)	Promotion of angiogenesis, improvement of the microenvironment of MI, and reduced ventricular remodeling	[115]
Laponite/gelatin hydrogel	Stem cell-derived secretome	Myocardial infarction (permanent occlusion, rat)	Ejection fraction and cardiac output were improved and cardiac remodeling was reduced	[116]
Mesoporous silica-iron oxide nanoparticles	Insulin-like growth factor	Myocardial infarction (ischemia-reperfusion, mice)	Cardiac function indexes such as left ventricular ejection fraction were improved and ventricular remodeling was reduced	[117]
PLGA human cardiomyocytes patch	Fibroblast factor 1 and CHIR99021	Myocardial infarction (permanent occlusion, mice)	Reduced cell apoptosis and improved ventricular remodeling	[118]
PLGA-PEG nanoparticles	Liraglutide	Myocardial infarction (permanent occlusion, rat)	Reduced scar thickness and cardiac dilatation, reduced myocardial cell apoptosis, and improved ventricular remodeling apoptosis	[119]
Wet tissue adheres to the hydrogel	Resveratrol and mesenchymal stem cells	Myocardial infarction (permanent occlusion, rat)	Improved cardiac microenvironment, reduced myocardial cell apoptosis, and improved ventricular remodeling	[120]
Exosomes derived from bone mesenchymal stem cells	Peptide specific for cardiomyocytes	Myocardial infarction (ischemia-reperfusion, mice)	Ejection fraction and other indicators were improved and ventricular remodeling was reduced	[121]
Extracellular matrix composite hydrogel	Gold nanoparticles	Myocardial infarction (ischemia-reperfusion, mice)	Improved the ejection fraction, improved ventricular remodeling, and reduced the content of ROS	[122]
Composite hydrogel	TIMP-3, FGF-2, SDF-1α	Myocardial infarction (permanent occlusion, rat)	Reduced ventricular dilatation, improved ventricular remodeling and increased cardiomyocyte survival rate	[123]
Mesenchymal stem cell-derived extracellular vesicles	–	Myocardial infarction (ischemia reperfusion, mice)	Reduced myocardial cell apoptosis, improved myocardial fibrosis, and reduced ventricular remodeling	[124]
